# Populating preterm infants with probiotics

**DOI:** 10.1016/j.xcrm.2021.100224

**Published:** 2021-03-16

**Authors:** Matthew J. Dalby, Lindsay J. Hall

**Affiliations:** 1 Gut Microbes & Health, Quadram Institute Bioscience, Norwich Research Park, Norwich, UK; 2 Norwich Medical School, University of East Anglia, Norwich Research Park, Norwich, UK; 3 Chair of Intestinal Microbiome, ZIEL - Institute for Food & Health, School of Life Sciences, Technical University of Munich, Freising, Germany

## Abstract

A randomized placebo-controlled trial by Martí et al.^1^ shows that probiotic supplementation of premature infants can modulate the infant gut microbiota soon after birth.

## Main text

Premature infants are vulnerable to severe health conditions including necrotizing enterocolitis (NEC) and sepsis in their first weeks of life. These conditions are linked to disruptions in establishment of the early life gut microbiota and overgrowth of potentially pathogenic bacteria, which may be acquired from the hospital environment. The use of probiotics is therefore often proposed as a prophylactic strategy to beneficially modulate the preterm microbiota and reduce incidence of NEC. In this issue of *Cell Reports Medicine*, Martí et al. add to the evidence for using probiotics in extremely premature infants, born with a weight of less than 1000 g.^1^ This randomized placebo-controlled trial (RCT) included 132 preterm infants, a large number of infants for this type of study. The results from Martí et al. are consistent with previous research showing abnormal gut microbiota in preterm infants in the first months of life, which represents a very different ecology to that observed in full-term infants. The probiotic was detected in almost all infants in the intervention group and in only a handful of infants in the placebo group. The relative proportion of the probiotic bacteria *L. reuteri* was high at week 1 at 45%. This quickly fell to less than 1% by week 5 despite continued supplementation. As counts of *L. reuteri* remained relatively constant, this suggests that other bacteria were colonizing and persisting more effectively and “taking over.” The absence of *Lactobacillus* in the placebo group indicates that cross contamination was prevented, which has been observed in previous probiotic RCTs. A major advantage of this study is that it is a longitudinal RCT, and that the research team also confirmed the presence of the probiotic in the infant stool. Several previous research studies have shown benefits to supplementing preterm infants with *L. reuteri* DSM 17938 without investigating the microbial changes in the gut.^2^ Martí et al. significantly add to this research by showing the changes taking place within the gut microbiota.

The same team previously published clinical outcomes from this supplementation trial^3^ and while they did find a benefit to infant growth, they did not observe a reduction in severe outcomes including NEC, sepsis, or overall mortality. However, it is important to note that the study was not powered to determine a difference in these rates. A recent systematic review has shown an overall benefit of probiotics reducing these severe outcomes in premature infants, with multi-strain probiotics showing a greater benefit.^4^ Therefore, the use of a single strain may be a factor for the “lack” of significant long-term microbiome remodeling seen by Martí et al.^1^ Indeed, Lactobacilli are not typically present in large numbers but have been reported to be important initial colonizers, in the first week after birth. This initial colonization may be important for providing very early benefits, including altering the gut environment by reducing oxygen levels, thus impacting ecosystem structuring—in this study shown by a reduction in the potentially pathobiont microbiota families Enterobacteriaceae and Staphylococcaceae. The authors point out that stool samples are biased toward colonic bacteria, while *Lactobacillus* may play more of a role in the small intestine. Therefore, potentially including a probiotic species such as *Bifidobacterium* that can dominate the infant colon may be useful. Moreover, the diet available to microbial residents of the infant gut will be different in specific gut niches; simpler sugars abundant in the small intestine (also absorbed by the developing infant), while more complex sugars pass through to the colon. Therefore, although infants fed breast milk may be best suited for probiotics that include *Bifidobacterium* (which often encode specialized enzymatic machinery to metabolize these complex sugars), other infants that are fed formula, which includes simpler sugars and other dietary fiber, may better “feed” a genus like *Lactobacillus*. Interestingly, the authors did not see any “diet” effects in this study, as *L. reuteri* prevalence and abundance was not significantly altered between formula-feeding versus combined formula and breast milk-feeding versus exclusive breast milk-feeding infants. While the testing of single-strain probiotics is important to understand their individual effects in preterm infants, combining them into multi-strain probiotics with prebiotics (a symbiotic approach) may be necessary for greater colonization and longer-term microbiota changes.

Another question relates to the rationale for using a particular probiotic bacterium in preterm infants. Is the aim to replicate the normal infant microbiota? Notably, the origin of the strain *L. reuteri* DSM 17938 (used in this study) was reported to be isolated from breast milk,^5^ which may represent a “natural” strain for colonization (although it is unknown whether this originated from the maternal gut or from sharing maternal-infant skin and/or oral cavity microbiomes). However, due to the underdeveloped gut physiology in extremely low-birth-weight infants, the environment may not provide an ideal niche for even ecologically relevant probiotic strains.

While *Lactobacillus* as a genus is not typically considered a common member of infant gut microbiota in European and North American cohorts, a relatively recent study from India^6^ utilized an isolated *Lactobacillus* bacterium (*L. plantarum* ATCC-202195) from a healthy infant and used this strain to supplement at-risk infants. Notably, they observed reductions in sepsis and overall mortality in over 4000 children enrolled into the RCT. This suggests there is not a one-size-fits-all approach for probiotics and that endogenous microbial “inhabitants” may be better suited to colonize infants and provide associated health benefits than other commercially available probiotics, particularly in geographically diverse infant populations.

The use of probiotics for preterm infants is still patchy globally and more high-quality randomized controlled trial evidence, as presented in this study, is needed. These studies need to consider that probiotics are living organisms that enter an ecosystem competing with other bacteria, which in turn may provide colonization resistance against potentially pathogenic bacteria and shape the gut environment in beneficial ways. That normal bacterial residents of the infant gut are more likely to persist longer term and prosper in this environment is an important consideration, thus a more ecological consideration for strain choice, and what they can “eat” may help target more effective probiotic interventions. Martí et al. have shown how supplementation with *L. reuteri* colonizes the gut soon after birth, with this linked to improved growth.^1^ However, greater microbiome modulation through combinations of probiotics may be necessary to enhance protection from severe disease such as NEC.
